# Synergistic Combination of Quercetin and Mafosfamide in Treatment of Bladder Cancer Cells

**DOI:** 10.3390/molecules29215176

**Published:** 2024-10-31

**Authors:** Carmela Spagnuolo, Francesco Mautone, Anna Maria Iole Meola, Stefania Moccia, Giuseppe Di Lorenzo, Carlo Buonerba, Gian Luigi Russo

**Affiliations:** 1 National Research Council, Institute of Food Sciences, 83100 Avellino, Italy; francesco.mautone1998@gmail.com (F.M.); iolemeola@hotmail.com (A.M.I.M.); stefania.moccia@cnr.it (S.M.); gianluigi.russo@cnr.it (G.L.R.); 2 Oncology Unit, Hospital “Andrea Tortora”, ASL Salerno, 84016 Pagani, Italy; direttoreuocpagani@gmail.com; 3 Associazione O.R.A. ETS, Oncology Research Assistance, 84134 Salerno, Italy; carbuone@hotmail.com

**Keywords:** bladder cancer, quercetin, mafosfamide, apoptosis, autophagy, drug resistance

## Abstract

Bladder cancer, which has a rising incidence, is the 10th most common cancer. The transitional cell carcinoma histotype is aggressive and often current therapies are ineffective. We investigated the anti-proliferative effect of quercetin, a natural flavonoid, in combination with the alkylating agent mafosfamide (MFA) on two human bladder cancer cell lines, namely RT112 and J82, representing the progression from low-grade to high-grade tumors, respectively. In both cell types, the combined treatment led to a synergic reduction in cell viability confirmed by a combination index of less than one, though different biological responses were noted. In J82 cells, MFA alone and, to a lesser extent, with quercetin caused cell cycle arrest in the G2/M phase, but only the combined treatment triggered apoptotic cell death. In contrast, in RT112 cells, quercetin induced autophagy, evidenced by the autophagosome formation and the increase in LC-3 lipidation. Interestingly, the synergistic effect was observed only when cells were pre-treated with MFA for 24 h before adding quercetin, not in the reverse order. This suggests that quercetin may help overcome MFA resistance to apoptosis. Although further studies are needed, investigating the combined effects of quercetin and MFA could help elucidate the mechanisms of drug resistance in bladder cancer treatment.

## 1. Introduction

Among all the cancers detected in men worldwide, urothelial carcinoma (UC) is the most diagnosed cancer of the urinary tract. Bladder cancer is a prevalent genitourinary malignancy with high recurrence and mortality rates [1]. Bladder cancer is responsible for an estimated 549,000 new cases and 200,000 deaths worldwide. These numbers highlight the disease’s significant global impact. According to the World Health Organization, men are four times more likely to succumb to bladder cancer than women, with incidence and mortality rates of 9.6 and 3.2 per 100,000, respectively [2,3,4]. Most urothelial cancers originate from the mucosa and are classified as “non-muscle invasive bladder cancers” if they do not invade the muscle layer. Conversely, “muscle-invasive bladder cancer” (MIBC) refers to cases where the cancer invades the muscle layers and the entire bladder wall [5,6]. Approximately 70–75% of newly diagnosed UCs are non-invasive or low-grade UCs [7]. Identifying these variants is crucial for accurate diagnosis and informed clinical decision-making. Understanding these different variants can help clinicians tailor treatments, leading to improved patient outcomes. In fact, their accurate identification is crucial for risk stratification, these variants being associated with a different grade of aggressiveness. For this reason, the development of new cellular models resembling various clinical variants of bladder cancer, with differing levels of aggressiveness and resistance to chemotherapy, poses a significant challenge for improving future treatment outcomes [8].

Standard cancer therapy is determined by various factors, including the type, grade, and stage of the disease, and typically includes multiple approaches such as surgery, intravesical chemotherapy, systemic chemotherapy, radiation therapy, immunotherapy, and/or targeted therapy, with consideration of the patient’s overall health. Currently, MIBC is treated with cisplatin-based neoadjuvant chemotherapy, which has been associated with improved survival outcomes [9,10]. A significant advancement in this area is also related to the approval of the anti-PD-L1 antibody avelumab as a maintenance therapy for patients who do not experience disease progression following platinum-based chemotherapy, leading to improved survival rates [11].

Additionally, an observational study demonstrated the efficacy of metronomic single-agent cyclophosphamide in advanced lines of treatment. The study found that in 16 patients, third-line chemotherapy with cyclophosphamide was associated with a progression-free survival of 18 (13–22) weeks and an overall survival of 38 (33–41) weeks [12]. Cyclophosphamide was also employed in combination therapy. In 42 patients with advanced UC after gemcitabine–cisplatin failure, metronomic oral cyclophosphamide combined with paclitaxel has been found to be safe and effective as salvage chemotherapy [13].

Cyclophosphamide is a nitrogen mustard belonging to the oxazophorine group; as an alkylanting agent, its mechanism of action consists in the formation of cross-links between DNA molecules in order to alter their synthesis and induce cell death [14]. Its primary toxicity affects the hematopoietic system and urinary tract. Cyclophosphamide requires conversion to its active derivative, 4-hydroxy-cyclophosphamide by cytochromes CYP 2B6, 2C9, and 3A4. Additionally, acrolein, a byproduct of its metabolism, is highly toxic to the bladder [14,15]. One synthetic analogue of cyclophosphamide is represented by mafosfamide (MFA), which, unlike the original compound, does not require hepatic activation to generate its active metabolite, as it spontaneously degrades to 4-hydroxy-cyclophosphamide [16]. The effects of MFA on various types of cancer cells have been assessed in preclinical studies and clinical trials [16]. In breast cancer, the efficacy of metronomic vinorelbine and MFA is influenced by insulin sensitivity [17]. However, limited data are available on its efficacy against bladder cancer [18].

Quercetin, a naturally occurring flavonoid, has garnered significant attention in cancer research due to its diverse pharmacological properties [19,20]. Several authors demonstrated the anti-tumoral effects of quercetin on bladder cancer cells, exerted by the activation of the apoptotic process, autophagy, or growth inhibition [21,22,23,24,25]. More recently, a comprehensive review summarized the cellular and molecular mechanisms triggered by quercetin in preclinical models resembling bladder cancer [26].

It is important to note that no clinical trials are currently investigating the therapeutic efficacy of quercetin in bladder cancer (https://clinicaltrials.gov/). However, interest in this compound against this type of malignancy remains strong, as shown by efforts to use chemical analogs of quercetin [27] or to deliver the molecule via nanostructures to enhance its bioavailability [28].

The rationale for the present work stems from a study published a few years ago that analyzed the efficacy of cyclophosphamide combined with quercetin in patients with metastatic bladder cancer suffering from grade 2 fatigue. After 100 mg/day oral cyclophosphamide plus 2 g/day oral quercetin, a complete and prolonged radiologic response was achieved with minimal toxicity [29]. This study showed for the first time that a combination of metronomic cyclophosphamide plus quercetin may have additive or synergistic effects in reducing both fatigue and disease aggressiveness with an excellent safety profile. This observation stimulated a deeper investigation into the molecular mechanisms that could explain the efficacy of the combined treatment in different bladder cancer variants characterized by varying degrees of aggressiveness. Proof-of-concept, robust cellular models, specifically RT112 and J82, were selected to represent the progression from low-grade to high-grade tumors.

The potential for quercetin and other phytochemicals to mitigate the adverse side effects commonly associated with chemotherapy is not new, as recently reviewed [30,31,32]. In addition, recent studies have indicated that the combination of flavonoids and chemotherapeutic agents can lead to enhanced therapeutic outcomes by modulating various cellular pathways involved in cancer cell proliferation and apoptosis [33,34]. The case of quercetin is particularly interesting, as our research team demonstrated that, when combined with the BH3-mimetic drug ABT-737, quercetin can synergistically induce apoptosis in B-cells from patients with chemotherapy-resistant chronic lymphocytic leukemia. This effect occurs through the direct inhibition of two kinases, CK2 and PI3K, which activate Mcl-1, a pro-survival factor from the Bcl-2 family, via the PI3K/Akt pathway [35,36]. This observation positions quercetin as a promising candidate for synergistic treatment strategies in bladder cancer management, similar to findings that have emerged in the context of other cancer types. Additionally, given quercetin’s ability to sensitize cells to apoptosis when combined with conventional pro-apoptotic drugs [20], we focused our bladder cancer study on the combination of quercetin and MFA, due to the well-established pro-apoptotic efficacy of MFA [37].

Quercetin was chosen as a candidate to study its synergistic effects with chemotherapeutic drugs in bladder cancer due to its ability to trigger autophagy [38]. Autophagy is a cellular process that degrades and recycles damaged organelles, proteins, and other cellular components to maintain homeostasis and respond to stress [39]. It plays a complex role in cancer, acting as a double-edged sword. In the early stages of cancer, autophagy can help suppress tumor formation by removing damaged organelles and proteins that may contribute to genomic instability and promote cancer cell survival. However, in established tumors, autophagy can facilitate cancer progression by allowing cells to survive under stressful conditions, such as low nutrient availability or hypoxia, by providing energy and building blocks through the recycling of cellular components. This can result in increased tumor growth and resistance to chemotherapy [40,41].

There is circumstantial evidence suggesting a connection between autophagy, quercetin, and bladder cancer. Recent studies indicate that autophagy inhibitors in bladder cancer can enhance tumor sensitivity to chemotherapy and radiotherapy, as demonstrated in preclinical research (reviewed in [42]), and quercetin, in particular conditions, can either inhibit autophagy to enhance apoptotic cell death or induce autophagic cell death in tumor cells [38].

In the present work, we have delved into the analysis of the anti-proliferative effect of quercetin in combination with the alkylating agent MFA on two human bladder cancer cell lines, namely RT112 and J82, representing, respectively, the progression from low-grade to high-grade tumors. Notably, we examined the synergistic effect in the context of understanding the mechanisms related to drug resistance in bladder cancer treatment.

## 2. Results

### 2.1. Quercetin Enhances the Cytotoxic Effect of MFA in Bladder Cancer Cells

**Figure 1:**
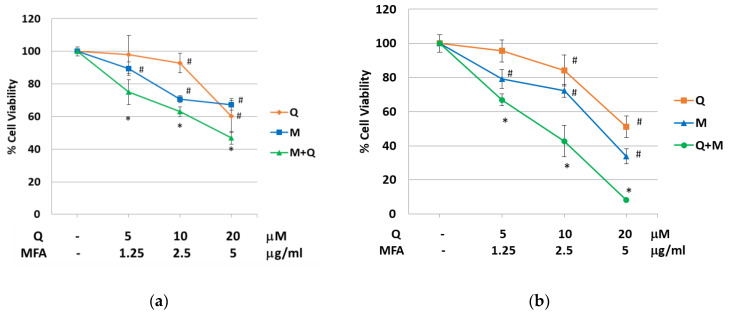
Quercetin enhances the cytotoxic effect of MFA in RT112 and J82 cells. RT112 (**a**) and J82 (**b**) cells were treated with mafosfamide (M), quercetin (Q), or their combination, as indicated. Cell viability was evaluated by crystal violet assay after 48 h of treatment. Line graphs represent the mean of n = 3 experiments performed in triplicate (s.d.). Symbols indicate significance: *p* < 0.05 (#) with respect to untreated cells and *p* < 0.05 (*) with respect to Q and MFA mono-treatments.

The RT112 and J82 bladder cancer cell lines used in this study represent low- and high-grade tumors, respectively. Figure 1 illustrates a dose-dependent reduction in cell viability following 48 h treatment with increasing concentrations of the single alkylating agent MFA or quercetin. The IC_50_ values (inhibitory concentration) in RT112 cells were 24.4 µM for quercetin and 8.55 µg/mL for MFA. In J82 cells, the calculated IC_50_ values corresponded to 21.8 μM for quercetin and 3.61 μg/mL for MFA.

To determine the concentrations of quercetin and MFA to use for the combined treatments, two factors were considered: i. the low bioavailability of the flavonoid, which at best reaches a plasma concentration in the low micromolar range; ii. the need to minimize toxicity by applying concentrations below the calculated IC_50_ values.

As reported in Figure 1, the combined treatment significantly enhanced the cytotoxic effect compared to the individual treatments in both cell lines. To assess if the combinatorial treatments induced a synergistic effect, the Combination Index (CI) was calculated using “CompuSyn” software. The Chou–Talalay method for drug combination allows for the quantitative determination of drug interactions, where CI < 1, =1, and >1 indicate synergism, additive effect, and antagonism, respectively. In J82 cells, the CI was calculated considering the combined administration of the two molecules at a constant (1:0.3) and non-constant ratio. As reported in Table 1, CI values were always less than 1, clearly indicating a synergistic effect.

Differently, in RT112, treating cells with the two molecules at a constant and non-constant ratio, the synergic effect (CI > 1) was measured for all combinations, except when quercetin and MFA was combined at higher concentrations where an antagonist effect was observed (CI > 1) (Table 2).

**Figure 2:**
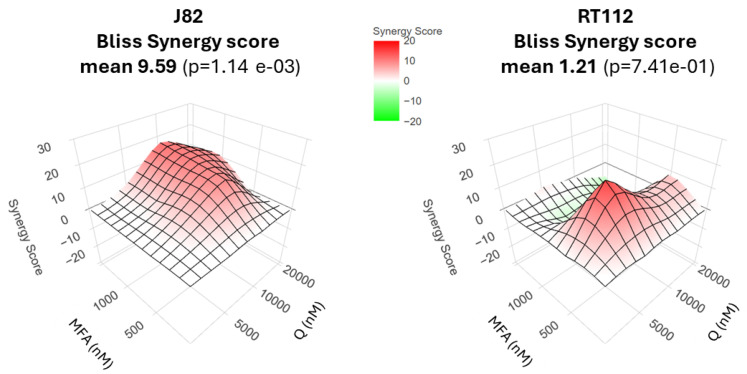
Bliss synergy score evaluation. In surface plots, red area indicates combination was judged synergistic more than antagonistic; green indicates combination was judged antagonistic more than synergistic.

To confirm the synergistic effect of the combinatorial treatment, data were also analyzed by the Bliss independence method. As shown in Figure 2, the Bliss score was 9.5 in J82 cells, indicating a strong synergism generated by the combination of MFA+Q. In RT112 cells, the Bliss score value was 1.21, indicating a synergistic effect, although less incisive, similar to the CI value (Table 2).

To determine whether the cytotoxicity associated with quercetin and MFA was limited to the malignant phenotype with respect to a model of normal cells, quercetin and MFA were tested alone and in combination on human lymphocytes isolated from peripheral blood. Data presented in Supplementary Figure S1 clearly indicate that, at the same concentrations applied in Figure 1, quercetin and MFA, either alone or in combination, only slightly reduced cell viability by about 15–20%, suggesting the higher sensitivity of bladder cancer cells to the treatment.

### 2.2. Quercetin and MFA Impact on Cell Cycle and Induce Apoptosis in J82 Cells

Variations in cellular morphology were noted in J82 cells incubated with the compounds under investigation (Supplementary Figure S2a). Stimulation with quercetin led to a reduction in cell density and a slight swelling of the cells, which, overall, remained comparable to the untreated cells. In contrast, treatment with MFA resulted in a significant decrease in cell number and pronounced cellular swelling characterized by notably enlarged nuclei. In the combined treatment, the morphological effects of MFA were more pronounced, leading to a substantial reduction in cell density. To investigate potential alteration in cell cycle progression resulting from the treatments, J82 cells were incubated with quercetin and MFA, either individually or in combination, for 36 h and at the end of the incubation period, they were stained with propidium iodide for acquisition by flow cytometry.

**Figure 3:**
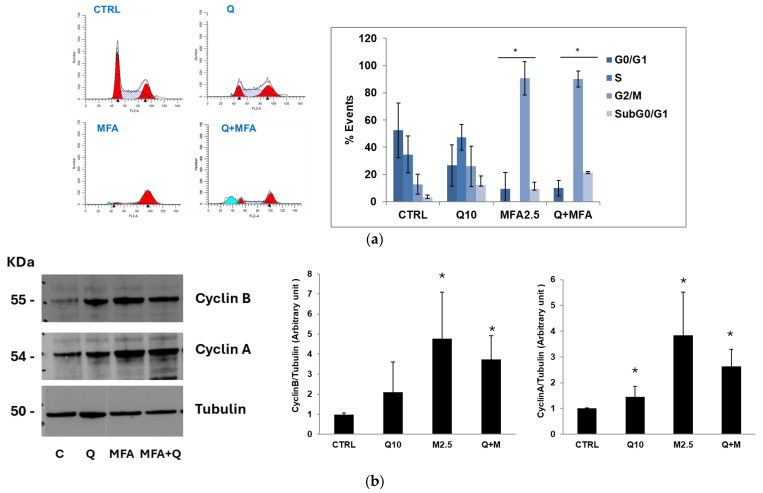
Quercetin and MFA’s impact on cell cycle in J82 cells. (**a**) J82 cells were stimulated with quercetin (Q; 10 μM) and mafosfamide (MFA 2.5 μg/mL) alone or in combination for 36 h and cell cycle analysis was performed using Modfit LT software. The average ± s.d. of the data obtained from n = 4 experiments, expressed as the number of cells (% events) present in the various phases of the cell cycle, is reported in the bar graph. Symbols indicate significance: *p* < 0.05 (*) compared to CTRL. (**b**) Immunoblotting analysis of cyclin B and cyclin A expression in treated J82 cells. Blots are representative of one out of n = 3 experiments for cyclin B and n = 2 experiments for cyclin A, performed separately. Densitometric analysis is reported in the bar graph on the left and is expressed as the ratio between cyclins and α-tubulin band intensities means ± s.d. Symbols indicate significance: *p* < 0.05 (*) compared to CTRL (C).

The representative histograms in Figure 3a indicate that treatment with quercetin alone caused an accumulation of cells in the S phase along with a slight increase in the G2/M phase. This suggests that quercetin may induce a block in the G2/M phase with cells gradually transitioning from S to G2/M. In contrast, treatment with MFA nearly completely arrested the cell population in the G2/M phase, a pattern also observed with the combined treatment. It is reasonable that MFA, as an alkylating agent, causes DNA damage, which triggers cell growth arrest in the G2/M phase through the ATM-p53 pathway [43].

To validate the flow cytometry analysis results, we examined the expression of proteins involved in the modulation of the cell cycle, specifically cyclin A, which plays a critical role in the S/G2 transition and cyclin B, a key regulator in the G2/M transition [44]. The cells were treated with the concentrations of quercetin and MFA used previously and incubated for 36 h. As shown in Figure 3b, these treatments led to an increase in the expression level of both cyclins. Quercetin treatment resulted in a two-fold increase in cyclin B compared to the control, while stimulation with MFA caused a five-fold increase. Cyclin A also rose, increasing 1.4-fold with quercetin and 3.8-fold with MFA compared to the control. The evident accumulation of these two key regulators of the cycle prevents cells from exiting the G2/M phase, leading to the observed cell cycle arrest.

**Figure 4:**
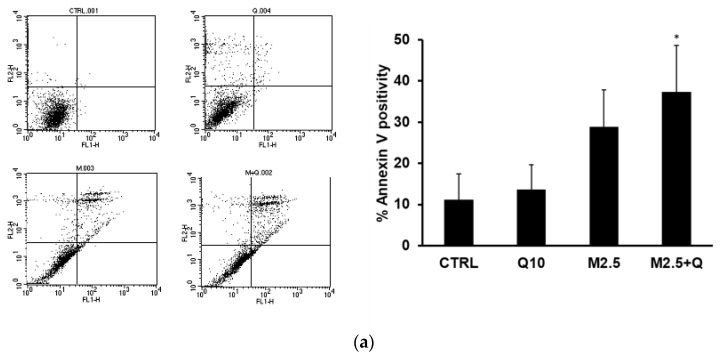

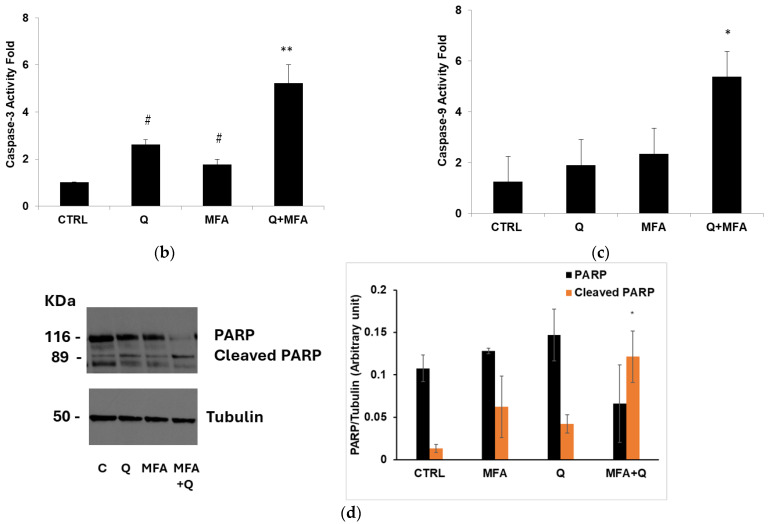
Quercetin in association with MFA induces apoptosis in J82 cells. Cells were treated for 36 h with quercetin (Q 10 μM) and mafosfamide (MFA 2.5 μg/mL) alone or in combination. (**a**) Annexin-V positivity was measured by cytofluorimetric analysis. Dot plots are representative of one out of n = 4 experiments. The proteolytic activity of caspase-3 (**b**) and caspase-9 (**c**) (nmol AFC/min/mg protein) was measured after 24 h of treatment. (**d**) Immunoblot showing the PARP cleavage. The bar graphs represent means ± s.d. derived from n = 3 separate experiments performed in duplicate. Symbols indicate significance: # *p* < 0.05 compared to CTRL, and *p* < 0.05 (*), *p* < 0.005 (**) compared to Q and MFA mono-treatments.

The flow cytometry analysis presented in Figure 3a indicates an increase in subG0/G1 peak when J82 cells were treated with MFA+Q, suggesting that the treatments induced cell death through the apoptotic process as a result of the cell cycle arrest. To confirm this hypothesis, specific assays were performed, including the evaluation of positivity to Annexin V and the activation of caspases 3 and 9, and PARP cleavage detection. One of the early events observed during the induction of apoptosis is the externalization of phosphatidylserine on the cytoplasmic membrane. J82 cells were incubated for 36 h with quercetin (10 μM) and MFA (2.5 μg/mL), both individually and in combination. At the end of the stimulation, positivity to Annexin V was measured by flow cytometric analysis. As reported in Figure 4a, quercetin and MFA induced an increase in Annexin V-positive cells of 1.34% and 16.9%, respectively, compared to the control. The combined treatment led to a significant increase in apoptosis of 26%, which was notably higher than that induced by either compound alone. To further confirm the activation of the apoptotic process, the activities of caspase 3 (effector caspase) and caspase 9 (initiator caspase) were also measured by incubating the cells for 24 h with quercetin and MFA, either alone or in combination. Consistent with the Annexin V results, the results in Figure 4b,c show that treatment with quercetin and MFA individually resulted in only a slight increase in caspase activity compared to the control, while the combined treatment resulted in a significant increase in enzymatic activities relative to both the control and the single-treatments. Additionally, the strong proteolytic cleavage of PARP, shown in the immunoblot in Figure 4d, provides further confirmation of apoptotic activity. Overall, our findings indicate that the combination of quercetin and MFA combination leads to a substantial reduction in cell viability through the activation of the intrinsic pathway that induces programmed cell death.

### 2.3. In RT112 Cells MFA Impacts on Cell Cycle While Quercetin Induces Autophagy

**Figure 5:**
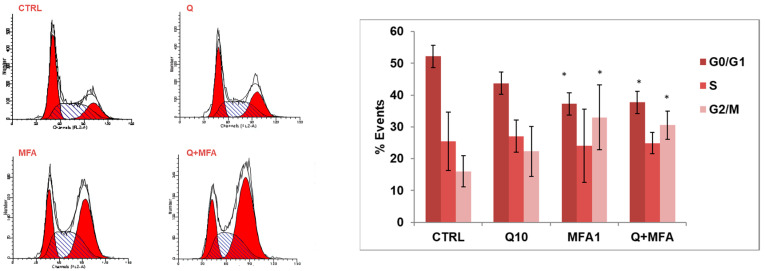
MFA and Q effect on the cell cycle in RT112 cells. Cells were stimulated with quercetin (Q 10 μM) and mafosfamide (MFA 2.5 μg/mL) alone or in combination for 36 h and cell cycle analysis was performed using Modfit LT software. The average ± s.d. of data obtained from n = 3 experiments, expressed as the number of cells (% events) present in the various phases of the cell cycle, was reported in the bar graph. Symbols indicate significance: * *p* < 0.05 compared to CTRL.

Cell cycle analysis in RT112 cells treated with MFA and quercetin showed that quercetin did not exert any significant effect, while the alkylating agent induced an arrest in the G2/M phase, also observed in the combined treatment (Figure 5).

**Figure 6:**
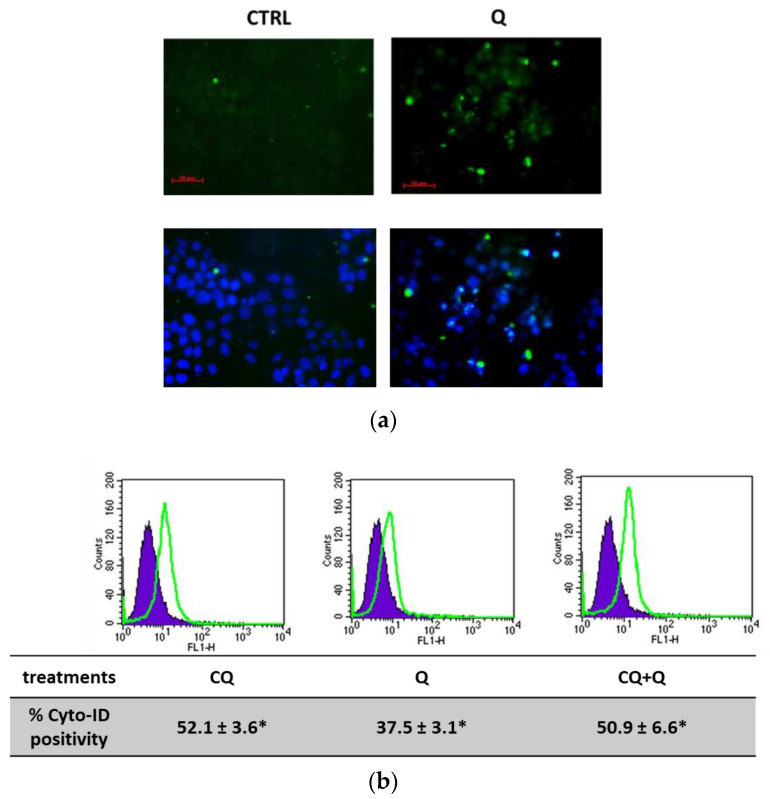

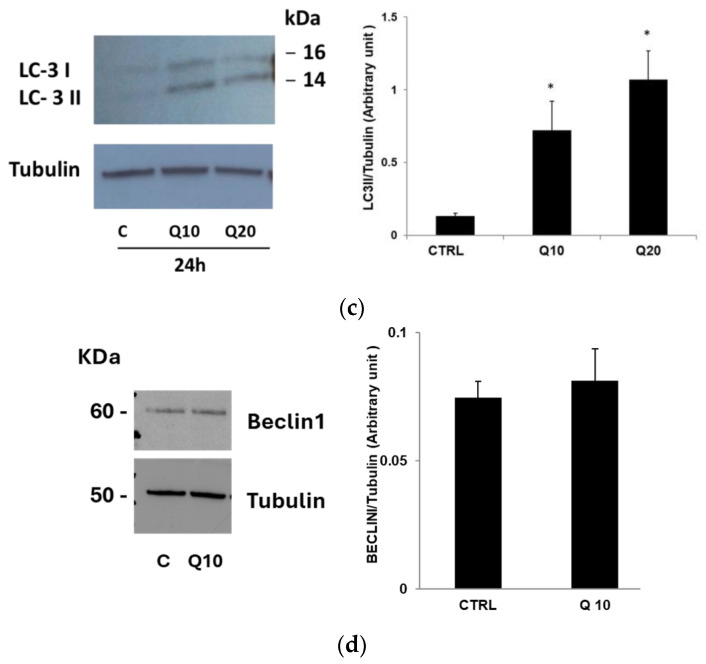
Quercetin induces autophagy in RT112 cells. (**a**) Representative images of autophagic vacuoles of cells untreated (left) and treated with 10 μM quercetin (Q) for 24 h visualized using a fluorescent microscopy and photographed in FITC/DAPI filters. (**b**) A representative histogram of CytoID flow cytometry acquisition of cells untreated (violet) and treated with 10 μM quercetin for 24 h (green line); the numbers below indicate means of n = 2 separate experiments ± s.d. (**c**) Immunoblotting analysis of LC3-I/LC3-II and (**d**) Beclin1 expression in RT112 cells treated with quercetin as indicated. Blots are representative of one out of n = 2 separate experiments performed. Densitometric analysis is reported in the bar graph on the left and is expressed as the ratio between LC3-II and the α-tubulin band intensities means ± s.d. Symbols indicate significance: *p* < 0.05 (*) compared to CTRL.

Microscopic observations indicated the presence of intracellular vacuoles in quercetin-treated RT112 cells (Supplementary Data Figure S2b), suggesting potential activation of an autophagic process. To investigate this hypothesis, several assays were performed to detect autophagy [45]. Following treatment with 10 μM quercetin for 24 h, RT112 cells were stained with Cyto-ID Green autophagy dye to visualize and quantify autophagosomes. As shown in Figure 6, this treatment resulted in a notable increase in fluorescent autophagic vacuoles, visible under fluorescence microscopy (Figure 6a) and quantified by flow cytometry, indicating a 37.5% increment compared to untreated cells (Figure 6b). Additionally, when the autophagic flux was inhibited using chloroquine (CQ), a pharmacological inhibitor, we observed an approximate increase of 50% in the presence of fluorescent vacuoles. Notably, the pre-treatment with CQ followed by quercetin did not cause a further increase in vacuole accumulation.

To further confirm autophagy activation by quercetin, we evaluated the expression of the molecular marker LC3-II, the lipidated isoform of the LC3 protein essential for autophagosome membrane formation. Immunoblots and the corresponding densitometric analysis presented in Figure 6c show a significant increase in LC3-II expression after 24 h of incubation with quercetin. In Figure 6d, moreover, we also measured a slight increase in Beclin 1 protein expression, an important autophagy regulator involved in autophagosome biogenesis.

**Figure 7:**
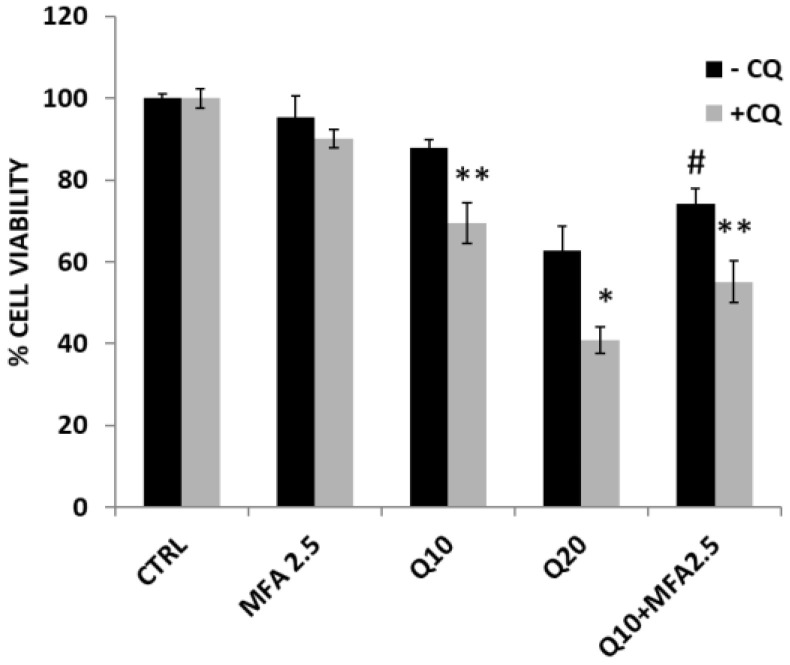
Quercetin induces a protective form of autophagy in RT112 cells. Black bars indicate cells treated for 24 h with MFA, quercetin (Q), or their combination, as indicated; the experimental point represented by gray bars were pre-incubated for 1 h with the autophagic inhibitor chloroquine (CQ 20 μM). Cell viability was evaluated by crystal violet assay. Bar graphs represent the mean of n = 3 experiments performed in triplicate (± s.d.). Symbols indicate significance: *p* < 0.05 (*) and *p* < 0.005 (**) compared to respective treatment without CQ; *p* < 0.05 (#) with respect to Q and MFA mono-treatments.

Additionally, we investigated which specific form of autophagy was induced by quercetin in RT112 cells. By ruling out cytotoxic and cytostatic autophagy, characterized by cell death and cell cycle arrest, respectively, both of which were undetectable after quercetin treatment (see Figure 5 and in Supplementary Figure S3), we aimed to determine whether the autophagy was protective or non-protective. To this end, if the autophagy was “protective,” we expected that inhibiting the autophagic flux with CQ would lead to increased cytotoxicity following quercetin treatment; conversely, in the case of “non-protective” autophagy, CQ would have no significant impact on quercetin’s cytotoxic effect. As shown in Figure 7, the pre-treatment with CQ followed by quercetin stimulation (gray bars) significantly reduced cell viability compared to quercetin treatment alone (black bars). Thus, we concluded that quercetin induces a protective autophagic phenotype in RT112 cells. Interestingly, following the pre-treatment with CQ and subsequent stimulation with either quercetin or the combination of quercetin and MFA, we observed an increased activation of the apoptotic process was observed, with an enhanced activation of caspase-3 activity and annexin V positivity (Supplementary Data Figure S3).

### 2.4. Quercetin Enhance Sensitivity to MFA in Bladder Cancer Cells

To understand the role of quercetin in enhancing the anti-proliferative activity of MFA in bladder cancer cells, we compared the effect on cell viability induced by the co-incubation of MFA and quercetin with those exerted when cells were pre-incubated with either quercetin or MFA.

**Figure 8:**
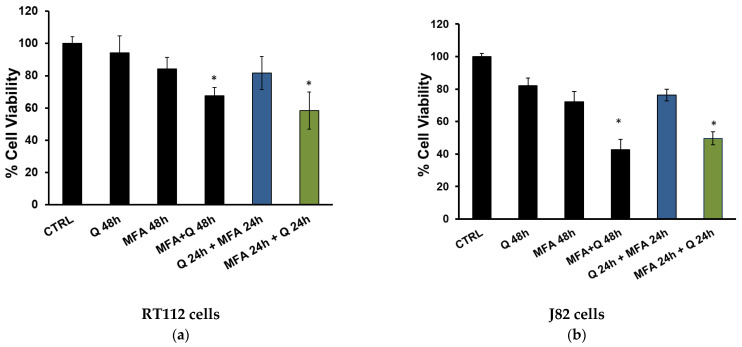
Quercetin enhances sensitivity to MFA treatment in RT112 (**a**) and J82 cells (**b**). Cells were treated with mafosfamide (MFA 2.5 μg/mL), quercetin (Q; 10 μM), or their combination, as indicated. Cell viability was evaluated by crystal violet assay after 48 h. Bar graphs represent the mean of n = 2 experiments performed in triplicate (s.d.). Symbols indicate significance: *p* < 0.05 (*) with respect to Q and MFA mono-treatments.

As shown in Figure 8, in both cell lines, pre-treating cells with MFA for 24 h before adding quercetin resulted in a synergistic effect (green bars), similar to that observed with co-incubation (black bars). However, when cells were pre-incubated with quercetin followed by MFA addition, the synergistic effect was lost (blue bars).

A key finding from our results is the observation that the combined treatment of MFA and quercetin enhances the sensitivity of RT112 (a) and J82 (b) cell lines to cell death.

## 3. Discussion

A rich, although controversial, body of literature suggests that combining flavonoids with chemotherapeutic agents may enhance therapeutic outcomes by modulating various cellular pathways involved in cancer cell proliferation and apoptosis, suggesting a promising area for further investigation in cancer treatment including bladder carcinoma [33]. Among flavonoids, quercetin has garnered significant attention in cancer research due to its diverse pharmacological properties. Known for its antioxidant and anti-inflammatory effects, quercetin may enhance the chemotherapeutic effects of drugs, like MFA, potentially addressing limitations in current bladder cancer treatment regimens [33].

Using two cell lines representing low- and high-grade tumors, RT112 and J82, respectively, our results demonstrate that combined treatment with quercetin and MFA leads to a significant and synergic reduction in cell viability across different phases of bladder cancer progression. This study serves as a proof-of-concept that natural compounds, when administered in adequate amounts and formulated to overcome bioavailability challenges, can enhance the therapeutic effects of conventional drugs, reducing their intrinsic cytotoxicity [46].

**Figure 9:**
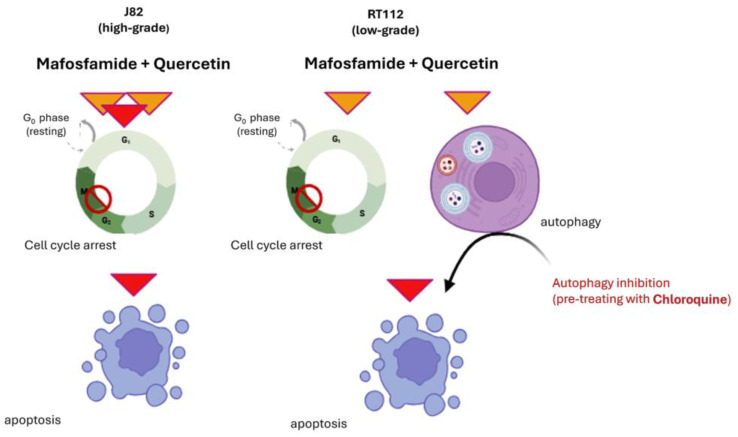
Graphical summary of the different responses to quercetin and MFA treatment in J82 and RT112 cells. Created with Biorender.com.

The biological response induced by quercetin varied between the two cell lines. In the RT112 cells, which are representative of low-grade bladder cancer, quercetin triggers a protective form of autophagy. In contrast, in J82 cells, which represent a high-grade stage, quercetin stimulation induced a cell cycle arrest, and when combined with MFA, led to apoptosis (Figure 9).

This variation in biological response is unsurprising, as a previous study from our team demonstrated that the phenolic extract from an Italian blend of extra virgin olive oil possessed chemopreventive potential and induced different forms of autophagy and apoptosis in the same human bladder cancer cell lines, depending on tumor progression [47].

Autophagy, a self-digestion process, has gained attention as a promising target in cancer therapy, particularly for bladder cancer marked by disrupted biological processes driving its progression. The role of autophagy in cancer is complex and context-dependent, acting as both a pro-survival and pro-death mechanism [48].

In bladder cancer, autophagy dysregulation is closely linked with cell death pathways; pro-survival autophagy can inhibit apoptosis and ferroptosis, while pro-death autophagy reduces tumor cell survival [49]. Autophagy’s influence on bladder cancer is multifaceted, affecting metastasis and interacting with the epithelial–mesenchymal transition (EMT) [49,50]. Pharmacological modulation of autophagy represents a promising strategy to slow cancer progression and enhance cell death. The pro-survival form of autophagy is an event that may occur with chemotherapeutic treatment, such as for cisplatin chemotherapy in bladder cancer cells [51]. In recent years, several chemical inhibitors for autophagy have been developed, and among these the CQ has been approved by the FDA (US Food and Drug Administration) for clinical use [52]. These autophagy regulators represent an important benefit in the case of protective autophagy, because the inhibition of autophagy can enhance the cytotoxicity of anti-tumor compounds [53]. This opportunity is also evidenced in our work where the pre-incubation with CQ significantly enhances the response of RT112 cells to quercetin and also to the combined treatment MFA plus quercetin (Figure 7 and Figure S3). Thus, our data strengthen the hypothesis that quercetin may act more effectively against carcinogenesis in association with specific autophagy inhibitors. Future investigations will focus on combining this natural compound with other autophagic inhibitors to enhance its therapeutic potential. In our model, we suppose that the low concentration of quercetin employed was unable to pass the threshold necessary to induce cell death, driving the cells into the limbo of autophagy, a condition that can evolve in opposite directions. In this condition, autophagy may either protect the cancer cells or contribute to their death, depending on the persistence of external treatment and the presence of additional stimuli.

The key lesson from this observation is the need to evaluate the potential anticancer effects of natural compounds with great caution, even when applied at low micromolar concentrations that are not typically cytotoxic. For example, quercetin’s ability to “protect” low-grade cancer cells (such as RT112) by inducing an autophagy mechanism that preserves cancer cell survival could inadvertently allow these cells to maintain their carcinogenic potential, an obviously undesirable effect. This emphasizes the necessity of carefully planned and targeted clinical trials to ensure both the efficacy and safety of nutraceutical interventions in cancer therapy [46].

In contrast, the data collected in J82 cells showed that the combination of quercetin and MFA induced cell cycle arrest in the G2/M phase, leading to apoptosis initiation. The combined effects of these compounds appear to impact the regulation of cell growth, likely by inhibiting the proteasomal degradation of cyclin subunits, thereby preventing the cells from entering mitosis. However, this does not lead to a robust induction of apoptosis or significant cell death when each compound is used individually suggesting that certain resistance mechanisms may arise with single treatments. This resistance seems to be overcome when quercetin and MFA are combined. As reported in Figure 9, quercetin, likely due to its pleiotropic activity, can “unlock” MFA-resistant cells and, therefore, induces a synergistic effect. Specifically, cells pre-incubated with MFA for 24 h before quercetin addition showed a marked reduction in cell viability compared to individual treatments. This effect was absent in cells pre-incubated with quercetin followed by MFA, suggesting that quercetin can influence molecular pathways that regulate the cell cycle and the apoptotic processes, possibly through the activation of the p53 pathway, a tumor suppressor activated in response to numerous stimuli such as DNA damage and oxidative stress. Additionally, quercetin might sensitize cells to MFA by shifting the balance between anti-apoptotic and pro-apoptotic proteins in favor of the latter. This natural compound could also modulate survival-related signal transduction pathways, potentially through the inhibition of protein kinases that drive these processes, either directly or indirectly.

The study of NF-κB activation in cisplatin-resistant bladder cancer cell lines demonstrated that cisplatin caused a marked induction of the transcriptional activity of this transcription factor [33,54,55]. Considering these findings, we can speculate that treatment with MFA may induce an inflammatory response likely mediated by NF-κB, which could lead to resistance to cell death. Therefore, the sensitizing capacity of quercetin might be due to its ability to directly or indirectly inactivate this transcriptional factor. This could occur by reducing NF-κB expression, preventing its nuclear translocation, activating the IkB inhibitor, enhancing its expression at the transcriptional and/or post-transcriptional level, or by preventing its degradation by the proteasome. To this extent, it is worthwhile to mention that the capacity of quercetin to negatively regulate the NF-κB pathway in several cellular settings has been previously described [56,57].

The potential use of quercetin combined with MFA in clinical settings, particularly to overcome chemotherapy resistance, holds significant promise. Chemotherapy resistance is a major challenge in treating many cancers, including bladder cancer. As reported in the Section 1, quercetin possesses the ability to sensitize cancer cells to apoptosis, possibly by modulating pathways like autophagy and inhibiting pro-survival proteins, enhancing the efficacy of chemotherapeutic agents like MFA. In preclinical studies, quercetin has shown a synergistic effect when combined with chemotherapy, increasing the sensitivity of cancer cells to treatment by overcoming resistance mechanisms [20,35,38]. This could lead to improved outcomes for patients with resistant tumors, increasing efficacy of previously ineffective treatments. However, translating these findings to clinical practice requires careful consideration. The optimal dosing and timing of quercetin administration, as well as its interaction with standard chemotherapy protocols, must be thoroughly investigated in clinical trials. Additionally, potential side effects, bioavailability limits (see below), and patient variability need to be assessed. If these challenges can be addressed, the combination of quercetin and MFA may represent an innovative approach to overcoming chemotherapy resistance and improving cancer treatment outcomes.

This study acknowledges the limitations commonly associated with the use of natural compounds for potential pharmacological applications, including anticancer activity. These issues have been extensively discussed in other studies [46,58,59]. One key concern is the very low bioavailability of quercetin, which makes it nearly impossible to achieve circulating concentrations of 10–20 µM in humans, even after oral administration of gram-level doses [60]. Therefore, the challenge remains in designing a clinical trial to assess the efficacy of the combined treatment of quercetin and MFA in patients with bladder cancer. Two solutions can be proposed. A previous phase I clinical study reported that the circulating concentration of free quercetin could be increased through intravenous administration of doses ranging from 60 to 1700 mg/m^2^. Following this treatment, individuals with cancer tolerated acute serum levels of 200–400 µM, indicating that quercetin can be safely administered via intravenous bolus, with plasma levels sufficient to show evidence of anti-tumor activity [61]. Alternatively, to avoid the need for intravenous infusion and use an oral formulation of quercetin, issues related to its poor solubility, bioavailability, and stability could be addressed by designing suitable nanocarriers to deliver the molecule. This area of research is highly active, and several promising quercetin nano-formulations have already been reported in the literature as potential strategies for tumor therapy [62,63].

An interesting possibility, although still speculative at this stage of knowledge, is the use of quercetin and MFA as intravesical therapy for bladder cancer, which represents an intriguing possibility for localized treatment. Intravesical therapy, which involves directly administering therapeutic agents into the bladder, allows for high drug concentrations at the tumor site while minimizing systemic side effects. This approach is commonly used in bladder cancer, particularly for early-stage or non-muscle invasive disease [64,65]. Quercetin could enhance the effectiveness of intravesical chemotherapy. MFA already has a proven ability to kill cancer cells, but its effectiveness is often limited by drug resistance. Combining these agents could offer a synergistic effect: quercetin might help overcome chemotherapy resistance by sensitizing the bladder cancer cells to MFA-induced cell death. Intravesical therapy would maximize quercetin and MFA’s contact with bladder cancer cells, potentially improving drug absorption and efficacy while limiting systemic exposure, thus reducing toxicity. However, challenges remain, including ensuring the stability and bioavailability of quercetin discussed above in the bladder environment, optimizing dosing regimens, and evaluating the potential for local toxicity or irritation. Further preclinical and clinical studies are needed to assess whether the combination can effectively enhance treatment outcomes in bladder cancer patients when delivered via this route.

In conclusion, our findings indicate that combining the alkylating agent MFA with quercetin, a natural molecule with a well-described pleiotropic mode of anticancer effects, could offer a promising therapeutic strategy for the treatment of urothelial bladder cancer. This combination not only enhances cytotoxic efficacy against cancer cells but also has the potential to reduce drug resistance. Future research should aim to elucidate the molecular pathways driving the synergistic anti-proliferative effects, to optimize and advance it towards clinical application.

## 4. Materials and Methods

### 4.1. Cell Culture and Viability Assessment

The cell lines employed in this study included RT112 (human bladder carcinoma epithelial cells) [66] and J82 (human urinary bladder transitional mesenchymal carcinoma cells) cells, both from the American Type Culture Collection (ATCC, Manassas, VA, USA) [67].

Cells were cultivated at 37 °C in a controlled humidified environment with 5% CO₂. RT112 cells were maintained in Roswell Park Memorial Institute (RPMI) medium while J82 cells were cultured in Minimum Essential Medium Eagle (EMEM) (Lonza, Euroclone SPA, Pero, Italy). Both media were enriched with 10% fetal bovine serum (FBS), 1% L-glutamine, and 1% penicillin/streptomycin (Lonza, Euroclone SPA, Pero, Italy). Cell viability was determined using a crystal violet staining method (Merck Life Science, Milan, Italy). Cells were plated in 48-well plates at a concentration of 8 × 10⁴ cells/mL and treated as outlined in the Section 2. After stimulation with quercetin (Merck Life Science) and/or MFA (Santa Cruz Biotechnology, Inc., Dallas, TX, USA), the cells were immobilized with 10% formalin for 10 min, rinsed, and subsequently treated with a 0.1% (*w*/*v*) crystal violet dye solution for 30 min. Subsequently, the stained cells were solubilized using 10% acetic acid, and absorbance readings were obtained at 590 nm using a spectrophotometer (Synergy HT microplate reader, BioTek, Milan, Italy).

### 4.2. Synergy Evaluation

The Combination Index (C.I.) was evaluated using the Chou–Talalay method for analyzing drug interactions, as previously described [68]. Dose–response curves were generated by determining the fraction affected (Fa), which indicates the percentage of cell death relative to untreated controls. Analysis of dose effects and calculations of the C.I. for the combined treatments were performed using CompuSyn software, which is available for free at www.combosyn.com.

Analysis using the Bliss independence method [69] was conducted with the freely available SynergyFinder+ (www.synergyfinder.org/#!/; access date: 25 October 2024) [70,71].

### 4.3. Autophagy Assessment

Autophagy was evaluated by measuring the presence of autophagic vacuoles and analyzing the expression levels of the lipidated form of the LC3-II protein.

#### 4.3.1. Quantification of Autophagic Vacuoles

Autophagy was monitored using the Cyto-ID Autophagy Detection Kit (ENZO Life Science, Milan, Italy) following the manufacturer’s guidelines. Briefly, after treatment, RT112 cells were washed and incubated with the Cyto-ID autophagy detection dye. Subsequently, the cells were rinsed with assay buffer, and autophagic vacuoles were visualized using a fluorescence microscope (Zeiss Axiovert 200, Carl Zeiss, Milan, Italy). Autophagosome quantification was performed through flow cytometry, equipped with a 488 nm argon laser and a 530 nm filter (FACS-Calibur; Becton Dickinson, Mountain View, CA, USA), and analyzed using Cell-Quest Pro software (Becton Dickinson, Mountain View, CA, USA).

#### 4.3.2. Western Blot Analysis

RT112 cells were treated as indicated, then collected and lysed using a buffer containing protease and phosphatase inhibitors, as previously described [72]. Protein concentrations were determined, and 30 µg of protein lysates were loaded onto a precast 4–12% Novex Bis-Tris gel (Life Technologies, Carlsbad, CA, USA) using MES (2-(N-morpholino) ethanesulfonic acid) buffer. Primary antibodies included anti-LC3 (cat #12741) and anti-Beclin1 (cat. # 4122S) (Cell Signaling Technology, Milan, Italy); anti-PARP (Santa Cruz Biotechnology, Heidelberg, Germany, cat #sc-8007); and anti-α-tubulin (Merck Life Science, Milan, Italy; cat #T9026). PVDF membranes were then incubated with horseradish peroxidase-conjugated secondary antibodies, followed by development using the ECL Plus Western blot detection system (GE Healthcare, Milan, Italy). Optical density was measured with a Gel Doc 2000 system (Bio-Rad Laboratories, Milan, Italy), and band intensities were analyzed using Multianalyst software 1.1 (Bio-Rad Laboratories, Milan, Italy).

### 4.4. Cell Cycle Evaluation

Following the specified treatments, J82 cells were collected, washed with PBS, and fixed in 70% ice-cold ethanol. The fixed cells were then washed twice with cold PBS and incubated with 50 µg/mL propidium iodide and 100 µg/mL RNase A (DNAse-free) in the dark for 1 h at 37 °C. Flow cytometry acquisition was performed using a FACSCalibur system (BD Biosciences, Sparks, MD, USA), and the cell cycle distribution was analyzed with ModFit LT software 3.0 (Verity Software House, Inc., Topsham, ME, USA).

### 4.5. Apoptotic Assays

The induction of apoptosis was assessed through specific assays, including Annexin V staining and measurements of caspase-9 and caspase-3 enzymatic activities.

#### 4.5.1. Annexin V Detection

Phosphatidylserine externalization was evaluated using fluorescein isothiocyanate-labeled (FITC) Annexin V (Miltenyi Biotec, Bologna, Italy). J82 cells, seeded at a density of 0.15 × 10^6^/mL, were treated and subsequently collected. After washing with PBS, cells were resuspended in 100 µL of binding buffer with 10 µL of FITC Annexin V and incubated in the dark at room temperature. Following incubation, cells were centrifuged and resuspended in 500 µL of binding buffer containing 25 µg/mL of propidium iodide. A total of 10,000 events were acquired using a FACSCalibur system (BD Biosciences), with low-fluorescence debris and necrotic cells excluded by gating before analysis using Cell-Quest software.

#### 4.5.2. Measurement of Caspase-9 and Caspase-3 Enzymatic Activities

To assess the enzymatic activities of caspase-9 and caspase-3, J82 cells (0.15 × 10^6^/mL) were treated as described in the Section 2. After treatment, the cells were collected, washed with PBS, and lysed in a buffer containing 10 mM HEPES (pH 7.4), 2 mM EDTA, 0.1% 3-((3-Cocamidopropyl)dimethylammonio)-1-propane sulfonate, 5 mM dithiothreitol, 1 mM phenylmethylsulfonyl fluoride, 10 µg/mL pepstatin A, 10 µg/mL aprotinin, and 20 µg/mL leupeptin. A total of 10 µg of cell lysate was added to the reaction buffer along with the corresponding amino-4-trifluoromethyl coumarin (AFC) substrates: Z-DEVD-AFC for caspase-3 and LEHD-AFC for caspase-9 and incubated at 37 °C for 30 min. The fluorescence of AFC was measured using a microplate reader (Synergy HT, BioTek) with an excitation wavelength of 395 ± 20 nm and an emission wavelength of 530 ± 20 nm. Enzymatic activities were quantified using an AFC standard curve, and caspase-specific activities were calculated as nmol of AFC/min/mg of protein at 37 °C with saturating substrate concentrations (50 µM).

### 4.6. Statistical Analysis

Data are presented as the mean ± standard deviation (s.d.) or mean ± standard error (s.e.), depending on the sample size. Student’s *t*-test was employed to determine the significance of differences between single treatments and controls, as well as between combination treatments and individual treatments.

## 5. Conclusions

In this study, we explored the anti-proliferative effect of quercetin in combination with the alkylating agent MFA on two human bladder cancer cell lines, namely RT112 and J82, representing the spectrum from low-grade to high-grade tumors. Our results demonstrate that this combined treatment induces a synergic reduction in cell viability eliciting distinct biological responses in each cell line. In J82 cells, we observed cell cycle arrest in the G2/M phase, leading to apoptosis. Conversely, in RT112 cells, quercetin triggered a protective form of autophagy, which was eventually overcome by the combined treatment with MFA.

While these findings warrant further investigation, the association between quercetin and MFA could provide valuable insights into the mechanisms underlying drug resistance in bladder cancer treatment. This study paves the way for future research focused on refining therapeutic strategies to effectively manage this malignancy.

## Figures and Tables

**Table 1:** Calculation of C.I. in the combined treatment of MFA plus Q in J82 cells.

Non-Constant Ratio (μM)	Fa ^1^	CI
Q10 + MFA0.1	0.35	0.7
Q10 + MFA0.5	0.38	0.8
Q10 + MFA1	0.49	0.8
Q10 + MFA5	0.88	0.4
**Total Dose Constant Ratio (MFA:Q 1:0.3)**	**Fa ^1^**	**CI**
0.94	0.1	0.78
4.7	0.5	0.77
16.9	0.85	0.86

^1^ Fraction affected (percentage of dead cells, for each experimental point).

**Table 2:** Calculation of C.I. in the combined treatment of MFA plus Q in RT112 cells.

Non-Constant Ratio (μM)	Fa ^1^	CI
Q10 + MFA0.1	0.15	0.8
Q10 + MFA0.5	0.22	0.9
Q10 + MFA1	0.30	0.9
Q10 + MFA5	0.38	2
**Total Dose Constant Ratio (MFA:Q 1:0.3)**	**Fa ^1^**	**CI**
0.25	0.1	0.22
1.75	0.25	0.85
4.05	0.35	1.54

^1^ Fraction affected (percentage of dead cells, for each experimental point).

## Data Availability

Data presented in this study are available on request from the corresponding author.

## Supplementary Materials

The following supporting information can be downloaded at: https://www.mdpi.com/article/10.3390/molecules29215176/s1, Figure S1. Quercetin and MFA effect on lymphocytes isolated from the peripheral blood of healthy volunteers. Figure S2. Morphological changes in J82 (a,b) and RT112 (c,d) cells treated with quercetin and MFA. Figure S3. Chloroquine pre-treatment increased the activation of the apoptotic process in RT112 cells treated with quercetin and MFA.
