# Supplementary material for: Synergistic Combination of Quercetin and Mafosfamide in Treatment of Bladder Cancer Cells
Source: Molecules. 2024 Oct 31;29(21):5176. doi: 10.3390/molecules29215176 (PMC11547860; doi:10.3390/molecules29215176)
Supplement: Supplementary file 1 [file molecules-29-05176-s001.zip › molecules-3217537-supplementary.pdf]

Article

Synergistic Combination of Quercetin and Mafosfamide in the Treatment of Bladder Cancer Cells

Carmela Spagnuolo [1,*], Francesco Mautone [1], Anna Maria Iole Meola [1], Stefania Moccia [1], Giuseppe Di Lorenzo [2], Carlo Buonerba [2], and Gian Luigi Russo [1]

SUPPLEMENTARY DATA

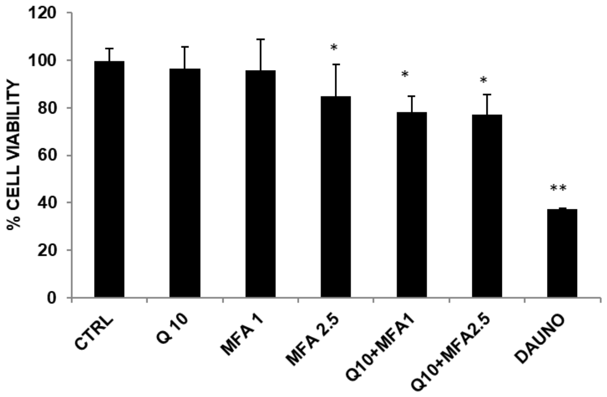

Figure S1. Quercetin and MFA effect on lymphocytes isolated from the peripheral blood of healthy volunteers. Lymphocytes from six healthy volunteers isolated by Ficoll reagent and cultured in RPMI medium supplemented with 10% FBS, 1% L-glutamine, and 1% penicillin/streptomycin. Cells were incubated for 48 h with various concentrations of the two single molecules or in association (quercetin: Q μM; mafosfamide: MFA μg/ml) and with Daunoblastine (Dauno 0.04 ng/ml) as positive control. Cell viability was determined using the Neutral Red assay and the percentage viability was calculated in relation to the control. Each value represents the average of six experiments performed in duplicate (± s.d.) with relative statistical significance (*=p <0.05; **=p<0.01 vs CTRL).

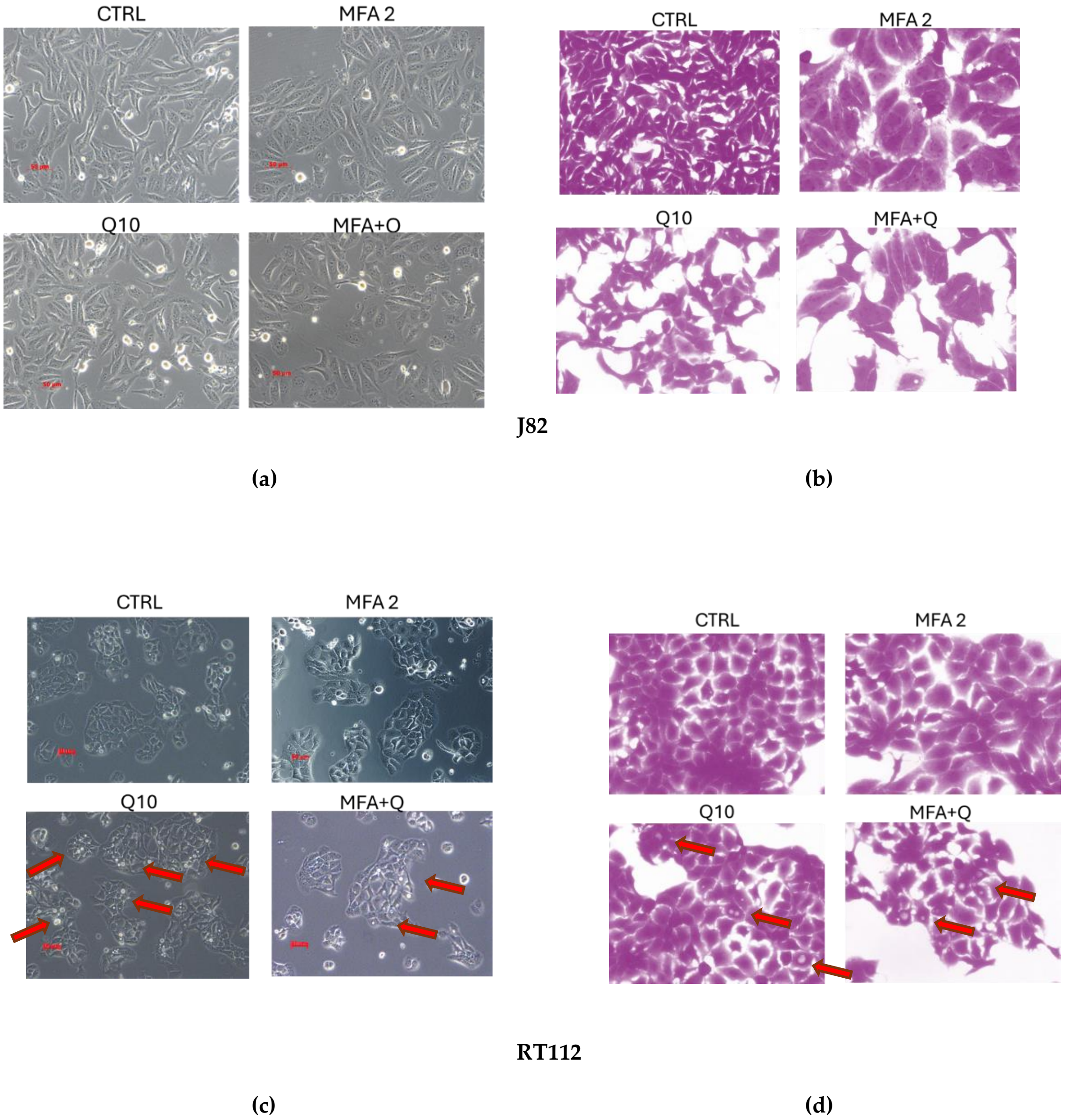

Figure S2. Morphological changes in J82 (a-b) and RT112 (c-d) cells treated with quercetin and MFA. Representative images of J82 and RT112 cells treated with quercetin (Q 10 µM), mafosfamide (MFA 2 µg/ml) and their combination for 48h. Unstained cells (a-c) and stained with crystal violet 0.05% solution (b-d) were photographed with optical microscope Axiovert 200 M Zeiss; 400x or 200x, in bright field. The arrows in panels (c-d) indicate the presence of intracellular vacuoles.

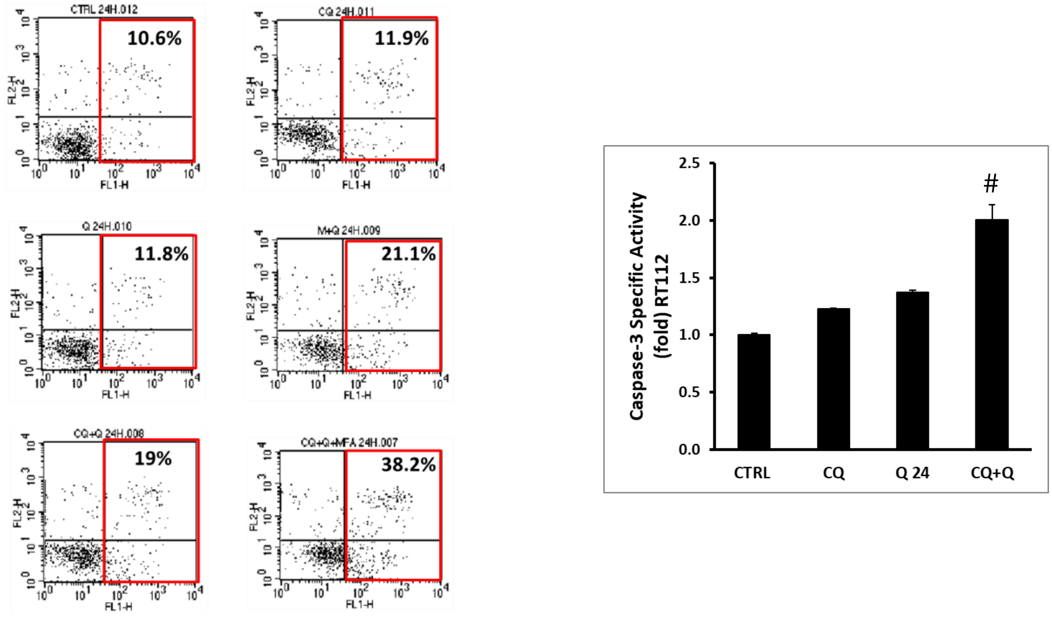

(a) (b)

Figure S3. Chloroquine pre-treatment increased the activation of the apoptotic process in RT112 cells treated with quercetin and MFA. Cells were pre-incubated for 1 h with the autophagic inhibitor chloroquine (CQ 20 μM) and successively treated for 24 h with quercetin (Q 10 μM) and mafosfamide (MFA 2.5μg/ml) alone or in combination. (a) Representative images of Annexin-V/propidium iodide positivity measured by cytofluorimetric analysis; numbers in the red rectangles indicate the percentage of Annexin V positive cells. In (b) the proteolytic activity of caspase-3 (nmol AFC/min/mg protein) was measured after 24 h of treatment. Bar graphs represent means ± s.d. derived from two separate experiments. Symbols indicate significance: # $p < 0.005$ compared to CQ and Q mono-treatments.
